# Annual Report of the Committee on Public Hygiene, Read before the Northern Medical Association of Philadelphia, Nov. 20th, 1851, by the Chairman, Wilson Jewell, M. D., and Ordered to Be Published

**Published:** 1852-01

**Authors:** 


### MEDICAL EXAMINER,

AND

### RECORD OF MEDICAL SCIENCE.

NEW SERIES.—NO. LXXXV.—JANUARY, 1852.

### ORIGINAL COMMUNICATIONS.

*Annual Report of the Committee on Public Hygiene, read before the Northern Medical Association of Philadelphia, Nov. 20th, 1851, by the Chairman,* WILSON JEWELL, M. D., *and ordered to be published.*

Life-knowledge, or that department of Medical Science which aims to prolong life to its natural limit, is the important and interesting field of inquiry, which has been assigned your Committee on Public Hygiene.

No subject possesses more intrinsic merit, than that which claims to develop the surest sources of health and life, and to secure "attainable longevity and exalted happiness." The sanitary question may justly be regarded, says Dr. Guy, of King's College, London, as "*the* great question of the day." Indeed, if we look at it as a question of humanity; or regard it as a great act of justice; or measure it by the rule of economy; or view it in its moral relations, we do not hesitate to say, that it has no rival.

Until of late years, public hygiene has elicited but little attention either in the halls of legislation or of science. In our own country it has been treated with great indifference, whether we view it in its physical, economical, or moral relations. Sanitary facts, for the most part, lie unrecorded. Statistical records, to show the waste of human life in cities over rural districts, have not been gathered. American authors, with but few exceptions, have been silent on the subject. American legislators have enacted laws upon every thing else than human health and life. With our profession it might be said truthfully, it has been an "unexplored region of medical inquiry;" while beyond its pale, there are only to be found a few solitary advocates for its claims upon the attention of men of science, or for its recognition by the public authorities, in order to meet the requirements of civilized life.

A brighter day, we trust, is dawning, as regards the importance of a watchful attention to the principles of public hygiene. Massachusetts has led the way, by making it a subject of scientific research, and by providing for a sanitary survey of the State. In our own city, the College of Physicians have been earnestly engaged in the prosecution of the science of Hygiene. One of the first acts of the American Medical Association, was the appointment of a Committee to report on Hygiene—thus opening a new and interesting door of scientific research for the profession, and laying a broad and deep foundation for its further and more enlarged prosecution.

Vital statistics and hygiene are twin brothers; they lie at the very threshold of the medical profession. Without a knowledge of these two important departments of medical science, no man can practice his profession well. Vital statistics give us an accurate account of births, marriages and deaths in certain districts and periods of time; the character of disease incident to certain professions or trades; a description of localities where disease has been most prevalent; the condition and vicissitudes of the seasons; the thermometrical, barometrical and hygrometrical changes that occur during the year; the prevalence of the winds, as also the geological formation of certain districts. Hygiene, on the other hand, is the hand-maid to medicine, and when properly understood and appreciated, is the means of saving thousands from an untimely grave. Hygiene is health; perfect health that condition of things which gives to its possessor strength, energy, power, buoyancy of spirits, and comprehends the perfect and harmonious performance of all the functions of the body.

How important, therefore, is the proper study and application of the sanitary science, or, in other words, of public health; what subject can transcend it? "We look upon things as valuable, that are worthless without life, and that cannot be enjoyed without health. How much more valuable, then, the means to possess and enjoy both life and health, which alone can give value to other objects!"

As medical men, members of a benevolent and God-like profession, the custodians of life and health, watchmen at the portals through which disease with its feverish and multiplied train of consequences enters, causing impaired health, physical debility, and oftentimes death among our citizens—it belongs to us to inquire into this great subject of public health—to discuss it, survey it, analyze it, and finally to preserve it, by the removal of numerous existing evils, the product of a neglect of the laws of hygiene. Furthermore, to suggest such measures as ought to be adopted for the improvement of public health, or, in one word, to remove the preventable causes of disease.

We are not of that number who look upon the medical profession as exclusively a curative one, and designed alone to alleviate the physical evils to which humanity is subject; having nothing to do with those great elementary principles, that determine the fullness of unalloyed health and length of days. On the other hand, we have always viewed our profession as conservative; that it belongs to us, as much to prevent disease, and to employ the means which our position and knowledge have placed within our reach, for the promotion and preservation of health, as to remove debility, to cure sickness or to heal injuries. How much better, how much more philosophical and philanthropic, to endeavor to arrest the march of disease, by a timely removal of its causes, appreciable only to the intelligent physician, than to wait, until they have penetrated and poisoned the fountains of life, and then attempt to repair the evil. We believe it is as much a part of our profession to teach the people how to avoid disease and how to live without being sick, as to cure them, when their systems have become undermined from any of the distressing maladies which "flesh is heir to."

In pursuing the subject of our report, we shall direct your attention more particularly to special Hygiene, and confine our remarks to some of the more prominent causes affecting individual and public health in our own city.

Philadelphia enjoys a high reputation, both at home and abroad, for its salubrity. What with its rectangular streets, its convenience for drainage, its natural advantages of surface and soil, the many excellent municipal regulations for cleanliness and the supply of pure and wholesome water, it is equal, perhaps, for health, to almost any city in the world. Had the design of its great founder been carried out in all its extent of detail, and had not the cupidity of property owners, whose only aim has been, to realize the greatest gain from their estates, been suffered to go on untramelled and unchecked by wholesome laws, we should at this day, have been in the enjoyment of a far greater immunity from disease. We are not wanting, however, in certain localities, where there exist fruitful causes for the deterioration of a pure and salubrious atmosphere, and the undermining of public health, which call loudly for removal; for, with the rapid increase of our own population and the continued influx of strangers from immigration, these causes are multiplying and extending, and in the same ratio with their increase, do we find a corresponding augmentation of disease and annual mortality.

The compass of a single report, however, will not allow of a careful examination into all the existing causes of insalubrity in and around our city. A few only of the more prominent must suffice.

Shattuck, in his admirable sanitary report to the Massachusetts legislature, recommends, that the causes of disease, should be divided into three general classes, viz: Atmospheric, Local, and Personal. The first, embraces all those agencies to which every person is alike exposed. The second, those to which persons living in a particular neighborhood or dwelling house are subject. The third, includes those which originate with the individual alone, independent of atmospheric or local causes.

Under the first head, or atmospheric, may be named climate, seasons, temperature of the atmosphere, its moisture, dryness, weight and composition, malaria, and those hidden conditions of the air, which have sometimes been called epidemic causes of disease.

Under the second head, or local causes, we may enumerate bad water, defective sewerage, drainage and surface cleaning, animal and vegetable effluvium, bad air, imperfect supply of light and heat, filthy or damp habitations, &c.

Under the third, or personal causes, are included hereditary constitution and imperfect organization, defective food, improper clothing, occupation, habits of life, exposure, excessive physical or mental exertion, &c.

Adopting the above division, we shall pass by the atmospherical and personal, and consider some of the principal local causes, for the insalubrity of our city, embracing bad or impure air, defective surface and sewer drainage, the construction of privies, and an inadequate supply of pure and wholesome water, especially for the laboring classes.

Impure air in our city, which may be considered as the result, to a great extent, of defective ventilation, both in many of our thoroughfares, narrow streets and alleys, in public and private houses, factories, schools, churches and prisons, is a most active cause of disease, and one of the principal, if not the chief agent, for the great amount of infant mortality which is so common in this and other large and crowded cities.

It is a well established physiological principle, that pure air is necessary for the maintenance of respiration, the changes in the blood, and the vitalization of all the tissues. Deprived of this *pabulum vitæ*, this great restorer and preserver of the exhausted animal frame, man sickens and dies. Deteriorate the air we breathe, and you lessen materially the chances of a long life ; hence is to be ascribed the greater mortality in cities over rural districts. In cities, the inhabitants inhale an atmosphere vitiated with impure and poisonous gases, and containing a less per centage of oxygen than the air of the country. An atmosphere thus impregnated, cannot be breathed with impunity, because man requires for the process of vitalization 24 cubic inches of oxygen every minute. Deprived of this supply, his health is impaired.

his nervous energy flags, his functions, both animal and mental fail to perform their accustomed offices with the vigorous tone of health, and his days are consequently shortened. It is remarked by statisticians that the difference of mortality between a city and a country life, is equal to forty per cent. in favor of the latter.

A pure atmosphere consists of one fifth of oxygen and four fifths of nitrogen, but when impure, we have it mixed with carbonic acid, carbonic oxide, sulphuretted hydrogen, ammoniacal and other deleterious gases. It is well known that man cannot breathe the same air twice with impunity, for the reason, that, during the process of respiration, it is deprived of a certain portion of its oxygen, and its place supplied by the carbonic acid given out from the lungs. Carbonic oxide, which is the product of imperfect combustion, and is often an ingredient in the atmosphere of cities and that of close apartments, is equally injurious in its action on the animal economy with carbonic acid gas. It contains a less proportion of oxygen, and cannot be inhaled even in a minute quantity without injury to health. Should the air we breathe contain only a 300th part of sulphuretted hydrogen and ammoniacal gases, which are frequent emanations from certain factories, slaughter houses, and whereever animal and vegetable matter is in a state of decomposition, we would soon become asphyxiated and die, or suffer in our health, in proportion as these or other noxious or poisonous gases are distributed in a less amount in the atmosphere.

It will not be denied that, in our own city, the atmosphere by which we are surrounded and which we are constantly inhaling, is impregnated, to a greater or lesser extent, according to circumstances, with exhalations from the lungs and bodies of men and animals, from decayed animal and vegetable matters, and from other noxious substances and gases. That the more imperfect the ventilation, the more defective the supply of oxygen, and, consequently, the more poisonous the air. It is on oxygen we rely for life and vigor, hence the importance of a free circulation of pure air in our streets and houses.

Wherever there is an over-crowded or excessive population in narrow or confined streets, or pent up courts and alleys, with small, contracted, and badly ventilated houses, with damp and foul cellars, and but a very imperfect supply of pure and wholesome water, there we find a deficiency of oxygen and a larger amount of noxious gases in the atmosphere. Is it any surprise that when epidemics prevail, they should be found in just such locations and in their most malignant forms? Should it be any cause of wonder, that cholera infantum selects its victims from the infant population of so foul a nursery for disease during the summer months? Nor can it be denied, that among the class of people who reside in our crowded and unventilated districts, or among those who are confined day after day in close work shops or factories, and who continually breathe an atmosphere deprived of its healthful amount of oxygen, we find scrofula and consumption in all their variety of forms to be prevalent diseases.

It is not, however, in the crowded dwellings of the poor alone, and in pent up courts, alleys and narrow streets, that we find defective ventilation. In the abodes of many whose circumstances and stations in society place them far above the wants of the common necessaries of life, there is a manifest lack of attention to, and appreciation of, healthful ventilation. No city, no street, no dwelling, no apartment whatever, can be really healthy unless a due regard is paid to ventilation; for it is an an essential condition to the comfort and well being of their inhabitants that they shall breathe a pure and wholesome atmosphere.

It is estimated, that one-third of the life of civilized man, is passed in apartments where there is a confined and deteriorated atmosphere, where, for the most part, open windows are precluded, and fires rendered either inconvenient or unnecessary as a source of warmth. Nor is it generally understood, how much suffering arises from the repeated inhalation of a vitiated air in such apartments. In public and private assembly rooms, in churches, courts of justice, offices, lecture and school rooms, work shops and factories, bed chambers, sick rooms, hospitals, theatres, &c., how much spent air must be taken into the lungs; "people who would revolt at the idea of drinking out of the same cup or glass with a stranger, or even with a guest, suffer no annoyance from, and feel no disgust at, inhaling what has already passed through the lungs of those who may be shut up in a room with them."

It is a source of regret that so little attention has been given to this vital subject, and it is high time that a proper system of ventilation in the construction of buildings, both public and private, should be made an object of primary consideration with those whose business is to draught architectural designs. It is evident that heretofore the builder or designer, as a general rule, has placed a far higher value upon the protection from without, and the beauty of arrangement within, in the construction of his edifice, than he has upon a free supply of pure air. It should, however, never be lost sight of, that ventilation, or an arrangement for a supply of air, both as regards quantity and quality, and the removal of that which has been vitiated, should be held as an essential requisite to every building, and form a leading feature in every design.

Whilst there has been considerable attention paid by our municipal authorities to street cleaning, drainage, sewerage, the cleaning of privies, the removal of local nuisances, and the enforcement of the same by suitable ordinances, some of the principal causes for the increased amount of annual mortality in our city, have been, in the opinion of your committee, entirely overlooked, viz: the proper construction of buildings; the building up of confined courts and alleys, without the necessary conveniencies for ventilation, light, privies and yard room; and their occupancy with a crowded population. It is really startling when we reflect upon the ignorance and indifference that is evinced in this particular respect by all classes in the community. Restrained neither by law nor ordinance, men build as they like, for convenience, for profit. They study and contrive how much they can realize out of their lots, by crowding them with houses that scarcely deserve the name, regardless alike of the misery and disease they entail on the unfortunate tenants. In too many instances, also, the tenants are heedless as to health and comfort in the selection of their residences, so they procure a cheap rent—but which, alas, may cost the loss of health, and perhaps of life, as the result of living in and breathing a vitiated atmosphere.

It is a clearly ascertained fact, that the inhabitants of densely populated and unventilated neighborhoods deteriorate in vitality. Dr. Simon, of London, has observed that: "Many, very many courts and alleys (in London,) hemmed in on all sides by high houses, having no possibility of any current of air; and worst of all, sometimes so constructed, back to back, as to forbid the advantage of double windows or back doors, and thus to render the house as perfectly a cul-de-sac out of the court, as the court is a cul-de-sac out of the next thoroughfare. It is surely superfluous to observe, that these localities are utterly incompatible with health. Among the dense population, it is rare to see any other appearance than that of squalid sickness and misery; and the children who are reproduced with the fertility of a rabbit warren, perish in early infancy. In the worst localities, probably not more than half the children born survive their fifth year."

The picture drawn by Dr. Simon of the condition of the population of London in the crowded and unventilated courts and alleys of that great metropolis, is by no means an unfair representation of what may be found in the crowded districts of our own city. It is well known that cholera infantum, dysentery, and many other diseases, select those neighborhoods where may be found a pent up and spent atmosphere. From the records of our Board of Health, it appears that there were 505 deaths from cholera infantum in 1850—319 of which were under one year, and the remainder within five years. Although we have no special records exhibiting the precise location of these fatal cases, still we are warranted in the conclusion that the majority of them occurred within the crowded districts. Dr. Isaac Parrish, in a valuable report he made in 1849, to the American Medical Association, says: "The amount of infant mortality in Philadelphia, believed to be traceable to a combination of heat, moisture and impure air in confined situations, is sufficient to excite anxious inquiry. The cause of a large portion of the cases of cholera infantum and other bowel diseases which annually carry off such a large number of children during the heat of summer, is as obvious as though it were tangible, and the cure often depends simply upon the removal of the patient from the crowded court or alley to the pure air of the country."

During the prevalence of epidemics, the inhabitants of the unventilated and crowded portions of a city suffer to a much greater extent than those in more airy and less crowded places. Nor can it be questioned, that these situations are *foci* for harboring and generating zymotic diseases. Evidence of this assertion may be found in the report of our Board of Health, during the prevalence of the cholera in 1849, in our own city, in which they say: "The increased ratio of cases to population in Southwark, must be attributed to its want of cleanliness, its locality, to the character of a portion of its inhabitants that reside in the more densely populated neighborhoods, and to its numerous confined and illy ventilated courts and alleys. That of Moyamensing, to the depraved condition of hundreds of its inhabitants, to the filthy and crowded state of many of its small houses or inhabited cellars, and their vitiated atmosphere."

In a report to the City Councils in 1849, we find some reference made to no less than ninety-one locations such as we have alluded to above, where ventilation and light are deficient, the buildings contracted, over crowded, and many of their inhabitants filthy and depraved. These ninety and one 'plague spots' are confined to the city proper; while in the Districts, especially in Moyamensing, Southwark, Northern Liberties and Kensington, we have a still larger number of badly ventilated courts, alleys and houses, with a crowded and degraded population. We could produce the most indubitable testimony that these localities are injurious to public health and productive of diseases of the lowest type. Dr. Parrish, in the same report we have already referred to, when alluding to precisely such neighborhoods, says, "We hazard nothing in saying that the seeds of scrofula and consumption are sown here; and the more acute diseases, which are distinctly traceable to a deficiency in the supply of the natural elements for sustaining and developing the frame, must prevail in these confined situations." Dr. Isaac Hays, in a letter to the Sanitary Committee of Councils in 1849, in reference to the health and cleanliness of the city, directs their attention to three points, as vastly important; one of which is the opening of all blind alleys and courts, and another, a law to prevent land holders occupying every inch of their ground with buildings, and placing privies in the cellars. The late Dr. Rush, in decribing the means of preventing summer and autumnal diseases, among other things, says, "In the construction of cities, narrow streets and alleys should be carefully avoided."

In 1666, when the inhabitants of London were venting their execrations upon a harmless bale of silks imported from Holland, as the vehicle of the seeds of the plague with which they had been afflicted annually for half a century, God mercifully relieved them from this dilemma, by permitting a fire to destroy whole streets and lanes of small wooden buildings, which had been the reservoirs of filth for centuries, and thereby the sources of all the plagues of that city. It is recorded that when a proposition was made to replace the burnt district by similar buildings, Sir Christopher Wren, who certainly entertained correct ideas of the advantages of ventilation to health in cities, opposed it, and said, "by so doing you will show that you have not deserved the late fire." They have not had the plague in London since that great conflagration.

A complete system of sewerage and drainage is of the first importance to the salubrity of a city and the comfort of its inhabitants. The relative health of those cities which enjoy the benefits of well constructed sewers and gutters, over those where none exist, is too palpable to require any comment.

The benefits of sewerage on the health of cities, is strikingly illustrated by Dr. T. Southwood Smith, before a Committee of the House of Commons, in which he says, "in every district in which the fever returns frequently and prevails extensively, there is uniformly bad sewerage."

The present system of draining by sewers in our city, while it is not in all respects what could be desired, has unquestionably proved itself useful in preserving health. It would seem, however, by a report from the City Surveyor in 1850, to the Committee on Highways, that an almost entire revision of the plan now in operation is required, before we shall attain that point of practical perfection in sewerage and drainage, by which the accumulating evils of the present imperfect system shall be avoided.

We have in operation at this time near thirty miles of under ground drainage through culverts, varying from three to ten feet in diameter in the clear. These sewers empty themselves into the Delaware and Schuylkill at various points.

The present method of ventilating and cleansing the sewers is exceedingly defective, and liable at times to render the atmosphere in the immediate vicinity of their inlets or their outlets into the docks, very unwholesome for breathing. The inlets from the streets where there are no traps, as in some of the districts, continually emit an offensive and sickening effluvium which is greatly increased in damp weather; while those arranged with traps, owing to the constant accumulation of offal in their mouths, make it necessary, every few weeks, to remove this deposit of mud and filth, which is oftentimes allowed to remain for several hours in the street, exposed to the sun's rays in the hottest days of summer, saturating the atmosphere around, with poisonous gases, highly injurious to health. A number of the city sewers are ventilated by means of iron pipes, planted on the pavement near the curb, elevated about four feet above the side walk, and pierced near and around their upper extremities with numerous small holes. This plan possesses no advantage, because, the foul air, escaping from the apertures, being somewhat heavier than the surrounding atmosphere, has difficulty in finding its way into the upper regions, and its accumulation becomes a source of much annoyance. Another plan lately proposed to carry off the foul air of the sewers, is, by the construction of lofty shafts or flues, elevated above the tops and built along side the walls of the houses. This method is favorably spoken of in England. We learn that one has already been erected in our city by way of trial, and promises to answer not only as a ventilator, but as a conductor for the noxious gases to a distance above the tops of the houses where they would be innocuous.

Another defect in our system of sewerage arises from the want of a uniform and extended plan in the regulation, not only of the grades but of the diameters of the culverts that have been laid down. In former years it would seem, that little attention had been bestowed upon progressive sewerage; the several culverts then constructed, were laid without a careful reference to the extent of surface required to be drained, to the natural features of the city plat, to the prospective increase of the city limits, or to heavy inundations from rains or sudden thaws, &c., the consequence of which is, that many of the old culverts being imperfect and unfitted, both as regards capacity and ventilation, in order to meet the increasing wants of those sections of the city in which they are located, contribute but a small share towards the comfort and health of the citizens.

Your Committee are gratified to learn that our intelligent City Surveyor has been for some time engaged in a searching survey of this whole subject, preparatory to the introduction of a complete system for the thorough drainage of the city. The speedy consummation of an object so important is really necessary, and it is to be hoped, that the wisdom and liberality of our Municipal authorities will not only favor this contemplated improvement, but will afford the Surveyor, every facility to carry out in detail, so noble, so philanthropic an enterprise, that will, when completed, contribute in a very large degree to improve the health of the community by a removal of one of the prolific causes of disease.

Surface drainage is adopted to a very great extent in our city, and where the gutters are kept in repair, with a sufficient descent from their summits to the inlets of the sewers, a proper supply of water to carry off their more solid contents, and the occasional use of the broom, they may not become a nuisance. But it cannot be denied, that in many of our streets, alleys and courts, either through negligence or from irregularity in the grading or an indifferent supply of water, there does exist oftentimes if not at all times, a collection of mud and offal that gives out a horrid stench, tending to deteriorate the atmosphere and produce sickness. During the hot months, this state of things is much increased, owing to the rapid decomposition continually going on wherever there are deposits of putrid animal and vegetable matter.

In some of these narrow and confined streets and alleys where resides a crowded and filthy population, the exhalations from the gutters are extremely nauseous and injurious to health.

Another evil from surface drainage is to be found in the mode of paving the streets with large rounded pebble stones; the interstices between each stone affording a nidus for mud and offal, which, decomposing under the rays of the sun during summer, become an aggravated cause for the encouragement of diseases. In addition to what we have advanced about surface drainage and its objectionable features, there is another grievous evil, arising from the mode pursued in street cleaning, which calls for reform. It is the practice of allowing street dirt, after being gathered into heaps, to remain for hours in the streets, and often in the hottest periods of the day. This refuse emits a poisonous exhalation, which is only increased whenever disturbed by the tramp of the horse or the carriage wheels, to the annoyance of those who pass that way.

That the emanations from sewers, gutters, streets, lanes and alleys, when badly paved and graded, or when not paved at all, are productive causes for disease in cities, no one in this enlightened age should, for a moment, deny. The evidence appended to the first report of the Sanitary Commission, appointed by Queen Victoria in 1844, in England, contains abundant proof of the correctness of this position. In their investigations of fifty towns, where the rate of mortality was the highest, and in these were included the largest manufacturing cities and the principal ports, after London—they say, that scarcely in one town in the fifty, was the drainage and sewerage complete; in seven it was indifferent, and in forty-two decidedly bad. The medical witnesses before these commissioners were unanimous in opinion, and brought facts to corroborate their assertions, "that no population is healthy which lives amid cess pools or upon a soil permeated by decomposing animal and vegetable refuse, giving off impurities to the air in their houses and streets." The commissioners also contrast the effects of the health of the inhabitants of sewered and unsewered towns. The duration of life is six years more in the sewered than in the unsewered streets of Ashton-under-Lyne. In Charlton, while the mortality in undrained streets amounts to four per cent. that in the drained districts is only two per cent. Dr. T. Southwood Smith, says, in the same report, that without attention to sewerage, all other precautions to preserve the health of towns must be in vain. Rotherhithe, a part of London, situate on the Thames, has been a favorite haunt of cholera. Its sewerage is deplorably defective, being by open sewers communicating with the Thames.

In the report of the cholera in 1849, in this city, by the Board of Health, it is observed that in Richmond district, the increased amount of disease when compared with other places, was believed to be owing "to its locality along the river front, its want of proper drainage and sewerage, &c." "In Kensington, to the unpaved, ungraded and undrained condition of many of its streets."

Dr. Isaac Hays, in his letter to Councils, in 1849, says, that "the entire abandonment of surface drainage" is important to the health of the city.

In the opinion of your Committee, surface drainage should not be allowed, wherever it is possible to dispense with it, by substituting underground drainage by means of a well constructed drain through iron pipes, leading from dwelling houses, with properly adjusted traps, and communicating with the sewers, which are to be found in our principal streets. Boston has very little surface drainage from houses, their gutters being dry, except after heavy rains. They send forth no offensive exhalation, and are perfectly innocuous.

We would also suggest the importance of having the street cleaning done before 9 A. M., or after 5 P. M., particularly during warm weather, and that all the year round, carts should follow in the wake of the scavengers, to remove the dirt and offal as rapidly as it is collected into heaps. These or similar sanitary arrangements, would no doubt add materially to the health of our city.

We have already intimated that Philadelphia is supplied with pure and wholesome water, and we may add, in greater abundance than that of other cities, obtaining their water in a similar manner, unless it may be Glasgow, where the supply is equal to 37 gallons per day to each citizen, and in New York, where the supply of water is said to be enormous. Our supply is about 30 gallons, per head, according to the report in 1849, of Frederick Graff, Esq., Superintendent of Fairmount Water Works. Although this may be considered an adequate supply for all personal purposes of life, yet your committee are of the opinion that it is not sufficient for more general and public purposes. Nor can it be repeated too often, that without an abundance of water, neither house nor street drainage can be perfect. With a scanty supply, kitchen offal and other refuse will accumulate, decomposition will take place in a few hours, in hot days especially, thus poisoning the atmosphere and becoming active causes for the formation and spread of disease.

Towards the poor of our city also, there is a short-sighted policy adopted by our public authorities as well as by landlords. They do not confer upon them those privileges and benefits arising out of the free supply of water for their domestic and personal use, which are enjoyed by their more fortunate neighbors. Few if any baths are to be found in the dwellings of the poor, and hydrant water for the necessary domestic purposes of the family is, in the generality of court houses, an article in great demand, owing to its limited supply.

On the subject of an ample supply of water, your committee would sugggest, that it only remains for the authorities, in order to render it among the most efficient means of public health in our city, to direct the daily opening of all the hydrant plugs, morning and evening, for half an hour during the summer months, and once a day through the spring and fall months, for the effectual cleaning of the gutters. In behalf of the poor, that public baths should be established for their use, and an ordinance be passed, requiring landlords and owners of property to introduce the Schuylkill water into every house, however small, for the use of the tenants. The benefit arising out of a liberal system, such as we have suggested, would soon be made apparent in the removal of much general and personal filth, prominent causes of disease; and be contributing to the moral as well as the physical improvement of the laboring classes in the community.

Another grave evil which mars the sanitary condition of our city, is to be found in the number and construction of privies. These may be considered as necessary evils, but they undoubtedly contribute in a large degree towards the production of disease.

It is estimated that our population of 409,000, would produce annually about 17,000 tons of night soil, and 130,000 tons of urine. Hundreds of privies in our city and districts, especially those attached to the houses in courts, and tenements in the smaller streets and alleys, are at all times full, or in a very offensive condition, notwithstanding the rigid supervision adopted by the Board of Health in order to prevent their becoming nuisances. Many of the privies are in near proximity to the dwellings or in the cellars, either for want of yard room or convenience, and emit the most fœtid exhalations, saturating, in some instances, the atmosphere of the whole house. In some courts, the inmates of the row, comprising a number of families, have only one or two privies in common.

No attention, in general, has been paid to the ventilation of privies. From this condition of things, can it be otherwise, than that the privies throughout our city are fertile sources of an impure atmosphere? Dwell one moment upon the fact, that 17,000 tons of human ordure, deposited in one year, is undergoing the process of fermentation and decomposition, exhaling their nauseous and poisonous gases into the atmosphere we breathe, contaminating it at our doors, and even in many of our dwellings, and the inference must be, that this evil is a prolific source for disease.

To determine upon a plan to dispose of this necessary refuse from a crowded population without its becoming a nuisance, would require more time and space than has been allotted to your committee in this report. The propriety of a change in the present system cannot be too strongly insisted upon, as it is an every day increasing and disgusting evil, in a sanitary point of view.

The present City Surveyor, has, in a report to Councils, already alluded to, suggested the plan, as far as practicable, of having the water closets to empty themselves into the common sewers, under certain regulations, which arrangement he thinks "would lead largely in future building to the introduction of water closets, in lieu of the old system of well privies, a system, nauseous in all its features, and more or less tending to inconvenience and disease."

Within a few days the Board of Health have given public notice, that all privy wells cleaned from and after December 1st, must be previously disinfected or deodorized. This we will allow is one step towards a change in the old method, and is an excellent sanitary measure, removing at once an intolerable nuisance during the process of emptying the wells; but it does not reach the evil to which we have alluded.

Before concluding this report, there is another subject, of some interest, to which your committee would briefly refer, to wit: has the ratio of mortality decreased in our city, as a consequence of the sanitary improvements made by the corporations from year to year? While we believe that much of the present salubrity of Philadelphia depends upon the wholesome sanitary measures adopted by the municipal authorities, it is nevertheless true, that no material change has been effected as regards the ratio of deaths to population. By a reference to Dr. Emerson's tables as published in the Amer. Jour. of Medical Sciences for several decennial periods, it will be found that the proportion of deaths to population has been as follows:

| | |
|---|---|
| From 1807 to 1820, a period of 13 years, there were deaths to population as - - - - - - | 1 in 47.86 |
| " 1820 to 1830, a period of 10 years, - - - - - | 1 in 38.85 |
| " 1830 to 1840, " " - - - - - | 1 in 41.15 |
| " 1840 to 1848, " 8* " - - - - - | 1 in 39. |

Figures are facts, and by these tables it will at a glance be seen, that the ratio of deaths to population has rather increased than otherwise. In conclusion, your Committee would say, that while the sanitary measures which have been adopted, consisting mainly of grading, paving, sewerage, cleansing the streets and privies, the removal of local nuisances and the present supply of pure and wholesome water, are advantageous to the health of the city, they are not adequate to the prevention of disease. Some of the most prominent causes have been overlooked; such as the practice of erecting small unventilated tenements in contracted streets, alleys, and blind courts, and overcrowding them with a filthy population; offensive and unwholesome factories, burial grounds, slaughter houses, hog pens, &c. While these things are allowed to remain in the heart of the populated districts undisturbed, and while in the absence of a better devised plan for the disposal of human ordure, the want of a more liberal supply of pure water for the streets and drains, but more especially for the houses of the laboring classes, together with public baths for the accommodation of the poor—our annual list of preventable diseases must not only continue to be large, but will increase; neither will the annual mortuary tables exhibit any favorable change in the ratio of deaths to population.

*This last octennial calculation is taken from a report of a Committee of the College of Physicians.

